# Auditory Electrophysiological and Perceptual Measures in Student Musicians with High Sound Exposure

**DOI:** 10.3390/diagnostics13050934

**Published:** 2023-03-01

**Authors:** Nilesh J. Washnik, Ishan Sunilkumar Bhatt, Alexander V. Sergeev, Prashanth Prabhu, Chandan Suresh

**Affiliations:** 1 Department of Communication Sciences and Disorders, Ohio University, Athens, OH 45701, USA; 2 Department of Communication Sciences and Disorders, University of Iowa, Iowa City, IA 52242, USA; 3 Department of Social and Public Health, Ohio University, Athens, OH 45701, USA; 4 All India Institute of Speech and Hearing (AIISH), Mysuru 570006, Karnataka, India; 5 Department of Communication Disorders, California State University, Los Angeles, CA 90032, USA

**Keywords:** noise-induced cochlear synaptopathy, noise exposure, speech-in-noise, auditory evoked potential, hidden hearing loss

## Abstract

This study aimed to determine (a) the influence of noise exposure background (NEB) on the peripheral and central auditory system functioning and (b) the influence of NEB on speech recognition in noise abilities in student musicians. Twenty non-musician students with self-reported low NEB and 18 student musicians with self-reported high NEB completed a battery of tests that consisted of physiological measures, including auditory brainstem responses (ABRs) at three different stimulus rates (11.3 Hz, 51.3 Hz, and 81.3 Hz), and P300, and behavioral measures including conventional and extended high-frequency audiometry, consonant–vowel nucleus–consonant (CNC) word test and AzBio sentence test for assessing speech perception in noise abilities at −9, −6, −3, 0, and +3 dB signal to noise ratios (SNRs). The NEB was negatively associated with performance on the CNC test at all five SNRs. A negative association was found between NEB and performance on the AzBio test at 0 dB SNR. No effect of NEB was found on the amplitude and latency of P300 and the ABR wave I amplitude. More investigations of larger datasets with different NEB and longitudinal measurements are needed to investigate the influence of NEB on word recognition in noise and to understand the specific cognitive processes contributing to the impact of NEB on word recognition in noise.

## 1. Introduction

Musical training involves processing auditory features in challenging situations while performing complex cognitive tasks [1]. Structured musical experiences can systematically shape the auditory–cognitive processes of professional musicians. Recent studies suggest that professional musicians have an “advantage” while processing suprathreshold sounds and can outperform non-musicians in a wide range of auditory perceptual tasks [2,3,4]. It is suggested that musical training can improve the coding precision of the acoustic features (e.g., frequency, intensity, rhythm, and duration) in the classical auditory pathway [5,6]. Moreover, musicians exhibit enhanced cognitive abilities, such as better attention and extended auditory working memory, which are necessary for auditory processing in background noise [7,8]. The musicians’ advantage is also reflected in auditory coding precision at the brainstem and cortical levels, measured through auditory evoked potentials [4,9,10,11]. 

A large body of literature indicates musicians’ advantage while processing speech in noise (SIN) [4,12,13]. Parbery-Clark et al. [4] studied the performance of age-matched young musicians and non-musicians with normal hearing sensitivity and similar non-verbal intelligence quotients. They reported that the performance of young musicians was significantly better than non-musicians in the QuickSIN and hearing in noise test (HINT), which are widely used clinical measures for assessing SIN perception. Musicians revealed better working memory (WM) capacity than non-musicians. The study further showed that WM was a significant predictor for QuickSIN scores, contributing to about one third of variability in the dependent variable. The number of years of musical training accounted for an additional 6% of the variability in the QuickSIN scores. Similar results were reported in older professional musicians when their speech-in-noise perception, WM, and auditory temporal acuity were compared to age-matched non-musicians [14]. Slater and Kraus [15] attributed the musicians’ advantage to heightened rhythm sensitivity, while other researchers attributed it to better pitch processing [12] and temporal resolution [16]. Taken together, recent evidence highlights that structural musical experiences can improve speech and music coding in the auditory pathway and improve auditory cognitive processes [17].

Professional musicians are routinely exposed to loud traumatic sound levels. A large body of literature suggests that hazardous sound levels encountered during musical training and performances can put them at higher risk of noise-induced hearing loss (NIHL) than non-musicians [18,19,20,21,22,23]. Collegiate students and music faculty members are exposed to sound levels that range from 80 dBA to 104.5 dBA during solo and group rehearsals and performances [24,25]. Gopal et al. [19] measured sound exposure levels among collegiate musicians during 50 min jazz ensemble activities and reported that the equivalent continuous noise level (Leq) during the ensemble ranged from 95 dBA to 105.8 dBA. Recent research indicates that about 40% of musicians report hearing difficulties due to high sound exposure [26]. Musicians have a 57% higher hazard ratio for tinnitus and an approximately four-fold higher hazard ratio for NIHL when compared to the general population [26]. In conclusion, recent evidence shows that professional musicians are at a higher risk of acquiring NIHL than their non-musical counterparts. 

NIHL is typically characterized by an audiometric notch at frequencies 3, 4, and 6 kHz with recovery at 8 kHz [27]. Phillips et al. [28] reported that 45% of young music students aged 18–25 showed a notched audiogram with 15 dB or greater notch depth in at least one ear. Recent studies suggest that conventional hearing thresholds are not sensitive enough to detect subtle hearing deficits induced by noise exposure, and a substantial amount of synaptic loss can remain “hidden” from behavioral audiograms [29]. Research on various animal models, including rodents [29], guinea pigs [30,31], and rhesus monkeys [32] have revealed that short-term exposure to medium to high-intensity noise can inflict irreversible damage to the synaptic connections between the inner hair cells (IHSs) of the cochlea and spiral ganglion neurons, even when hearing threshold recuperate and hair cell recovers. These studies examined the auditory functions before and after a complete recovery from temporary threshold shift (TTS) using distortion-product otoacoustic emissions (DPOAEs) and auditory brainstem responses (ABRs). The histopathological results of these studies showed abrupt and permanent loss of up to 50% of afferent nerve terminal connections between IHCs and auditory nerve fibers. The DPOAE amplitudes and ABR thresholds showed complete recovery to pre-noise exposure levels. However, ABR wave I amplitudes at high stimulus levels were significantly more reduced in noise-exposed animals than in the controls and the pre-exposure baseline. A similar loss of synaptic ribbons is observed in aging ears [33,34]. The loss of synaptic connections between inner hair cells and auditory nerve fibers without damaging hair cells or permanent threshold shift is referred to as noise-induced cochlear synaptopathy (NICS), also known as hidden hearing loss (HHL) [35]. Conventional hearing thresholds could substantially underestimate NICS [36]. There is evidence to suggest that the damage caused to auditory nerve fibers in NICS might also have functional consequences while processing suprathreshold stimuli, suggesting that suprathreshold measures might be more sensitive than conventional audiograms for detecting early staged NIHL [37,38,39]. 

In recent years, various attempts have been made to extend the findings of NICS in animal models to the normal-hearing human population. Recent studies on noise-induced HHL in humans showed a correlation between (1) auditory evoked potential measures and noise exposure history, (2) psychoacoustic measures and noise exposure, and (3) speech-in-noise measures and noise exposure history. Investigations in normal-hearing adults with high noise exposure have sometimes shown a correlation with ABR responses [40,41,42,43,44,45,46,47,48] and sometimes have not [49,50,51]. The studies showing an association between noise exposure and electrophysiological measures utilized different metrics of ABR. For example, Valderrama et al. [48] and Stamper and Johnson [46] showed a negative association between wave I amplitude and noise exposure; Grose et al. [41] showed reduced wave I/V amplitude ratio in the high noise exposure group; and Liberman et al. [43] reported an enhanced summating potential/action potential (SP/AP) ratio in their high-risk group. 

Recent studies examined the possible perceptual consequences of cochlear synaptopathy using psychoacoustic tests. Grose et al. [41] compared temporal and spectral modulation detection acuity and sensitivity to phase interaural phase differences between high- and low-risk groups of collegiate students. They found no significant differences between the groups on any psychoacoustic tests. Similarly, Fullgrabe et al. [52] investigated the influence of noise exposure on the ability to process temporal cues using a few psychoacoustic tests and found no significant difference between the high- and low-noise exposure groups on temporal acuity measures. 

In addition to ABR and psychoacoustic measures, researchers have used various speech-in-noise perception measures to study the functional changes associated with suspected primary neural degeneration due to high noise exposure history in individuals with normal hearing [41,43,48,49,53]. Liberman et al. [43] found that collegiate students at high risk of NIHL due to frequent exposure to noisy events/activities had poorer speech recognition in noise scores compared to age-matched controls. On the contrary, some studies did not find any significant association between speech-in-noise performance and noise exposure history [41,49,53,54]. 

Most investigations on cochlear synaptopathy in humans have focused on ABR measurements which provide information on the peripheral auditory system. In the present study, ABRs were recorded at low (11.3/s), medium (51.3/s), and high (81.3/s) rates, and at each rate, ABR wave amplitude and latency were obtained. ABRs obtained at low, medium, and high rates provide a way to look into the temporal dynamics of synaptic activity and neural conduction as the auditory system is strained [55]. High click rates are associated with prolonged ABR absolute and interpeak latencies and decreased ABR amplitudes [55]. The ABR protocol utilized in this study mainly targets the peripheral adaptive processes which capture the inefficiencies in synaptic processing associated with NICS. It is also important to consider that any damage to the peripheral auditory system may cause dysfunction in the central auditory system [56,57]. Hence, the influence of peripheral pathology such as NICS on the central auditory system could be examined by incorporating a test battery that includes tests sensitive enough to identify subtle electrophysiological changes in the peripheral and higher auditory centers and associated functional changes such as speech-in-noise perception.

The higher auditory centers can be analyzed using non-invasive long latency auditory evoked potentials such as P300. P300 is one of the auditory evoked late latency responses generated by the reticulothalamus, frontal cortex, and medial septal area and occurs approximately 250–350 ms after the stimulus onset. P300 is typically elicited by an instructed and infrequently presented target stimulus [58,59]. The P300 response is associated with stimulus assessment and allocation of attentional resources while updating working memory [60]. Thus, the inclusion of the P300 measure in the test battery for evaluating NICS can help in determining the influence of noise exposure on the central auditory system. 

The goal of this study was to determine (1) the effects of noise exposure on the peripheral and central auditory nervous system (CANS) functioning using electrophysiological measures among young musicians and non-musicians, and (2) the effects of noise exposure history on speech recognition in noise at the word level and sentence level among young musicians and non-musicians. To address these objectives, we evaluated ABR waveforms (waves I and V obtained at low, medium, and high stimulus repetition rates), P300 measures, and speech-in-noise performance in young musicians and non-musicians. 

## 2. Materials and Methods

### 2.1. Participants

The study was approved (IRB number—18-X-247) by Ohio University’s Institutional Review Board (IRB). A total of 38 students aged 18–30 years were enrolled from Ohio University’s School of Music and non-music disciplines. Student musicians were selected because of their routine exposure to loud sounds during ensemble and solo rehearsals, and their noise exposure is expected to be higher than non-musicians. Participants’ inclusion criteria were (a) no history of hearing, tinnitus, balance, or language impairments and (b) no history of previous developmental, cognitive, neurological, and attention-related disorders. All the participants were recruited via emails and flyers posted across the Ohio University campus. 

The enrolled participants were asked to complete an online noise exposure questionnaire [61], which would quantify their annual noise exposure background (NEB). The noise exposure questionnaire includes questions on the duration and frequency of noise exposure and provides a quantitative estimate of annual noise exposure. Based on the responses to the online questionnaire, 20 non-musicians (10 males and 10 females) and 18 musicians (11 males and 7 females) of European descent were shortlisted and recruited for the study. Participants of European descent were selected in this study, as previous investigations indicate that people of European ethnicity are more prone to NIHL than people of African ethnicity [62,63]. The recruited student musicians were percussion, brass, and saxophone majors. Participants were contacted via email to schedule appointments for the testing session. 

The data were collected in two sessions. The first session includes a brief case history, a battery of hearing tests, a consonant–vowel nucleus–consonant (CNC) test, and an AzBio sentence test. The second session was composed of DPOAE and electrophysiological (ABR and P300) tests. Both data collection sessions occurred within 15 days of each other.

### 2.2. Noise Exposure Questionnaire

Before the scheduling of appointments for the testing sessions, each participant’s noise exposure history was measured using an online noise exposure screening questionnaire. This noise exposure questionnaire was developed by Johnson et al. [61]. This questionnaire has been validated for estimating the overall annual acoustic exposure and used in previous investigations to quantify noise exposure in young adult populations [46,64,65].

Participants were required to submit their responses to the online questionnaire (Supplementary Material S1) at least a week before the first testing session. This questionnaire was used to estimate participants’ annual noise exposure background (NEB). The first part of the questionnaire has nine sections targeting different types of noise exposure such as aircraft, firearms, heavy equipment, power tools, music through speakers, and headphones. The second part of the questionnaire consisted of nine questions related to the musical instruments played by the participant. The questionnaire also includes questions on the duration and frequency of noise exposure. The participants’ responses were elicited using a forced-choice method and then rated by category to calculate the noise dose of last 12 months. The noise dose was calculated via these responses for each area of high noise exposure. Time spent in routine or mundane activities performed in quiet environments was calculated by subtracting overall time spent in noisy activities from 8760 h (365 days/year × 24 h/day). Questionnaire responses were further used to calculate the activity-related noise dose and overall annual noise dose, reported as *L_A_*_eq8760h_. Here, “*L*” represents the sound pressure level measured in decibels (dB), “*A*” indicates application of A-weighted frequency response; “eq” represents the sound pressure level (in dB) equivalent to the total acoustic energy over a given amount of time; and “8760 h” represents the overall duration of the noise exposure in hours over one year (365 days/year × 24 h/day). *L_A_*_eq8760h_ was extracted from the questionnaire responses utilizing the 3-dB exchange rate for calculation of the time/intensity level relation. Details of the questionnaire are reported in Stamper and Johnson [46] (2015) and Johnson et al. [61].

Non-musician participants who reported playing any instrument including voice on a daily basis were excluded from the non-musician group. For the purpose of this study, non-musician participants with *L_A_*_eq8760h_ values of 76 or greater were not included in the study. All the student musician participants had *L_A_*_eq8760h_ values higher than 76. Potential participants were contacted via email to schedule appointments for the testing sessions. Participants were also informed through email to avoid loud sound exposure for at least 12 h before the testing appointment time. Before administering the tests, the participants were asked to confirm that they had abstained from loud events or activities as requested. Participants who reported exposure to loud sounds in the last 12 h were rescheduled.

### 2.3. First Session

The session I started with obtaining informed consent, followed by a brief case history, which comprised questions related to health, hearing, head trauma, and balance. After completing the brief case history, an otoscopy was performed on both ears of each participant, followed by a middle ear examination (tympanometry), pure tone audiometry and extended high-frequency audiometry. Tympanograms of both ears were obtained over a pressure range of +400 to −400 daPa using a 226 Hz probe tone presented through a GSI 39 (GSI, Eden Prairie, MN, USA) middle ear analyzer. All the participants had a normal type “A” tympanogram. Hearing sensitivity was measured in an audiometric testing booth meeting ANSI standards (ANSI S3.1e1999). Air conduction thresholds were obtained for both ears at 250, 500, 1000, 2000, 3000, 4000, 6000, and 8000 Hz using an audiometer (AVANT MedRx, Largo, FL, USA) with ER-3A insert earphones (Etymotic Research. Inc., Elk Grove Village, IL, USA). The pure tone average of the hearing thresholds at 3000, 4000, and 6000 Hz (PTA346) was also calculated because hearing sensitivity at these frequencies is typically affected in individuals with high noise exposure history. Normal hearing of participants was defined as audiometric thresholds of ≤15 dBHL for frequencies between 0.5 and 8 kHz, and this was one of the inclusion criteria for the study. Extended high-frequency audiometry was carried out using circumaural earphones (Sennheiser, HDA 200) at 10, 12.5, and 16 kHz. At these extended high frequencies, the hearing thresholds were averaged to obtain the extended high-frequency pure-tone average (EHFPTA). 

After high frequency audiometry, word and sentence recognition in noise was tested binaurally using the CNC and AzBio tests, respectively. The CNC test assesses open-set monosyllabic word recognition in quiet and noise. A customized MATLAB program for controlling the stimulus presentation was utilized to administer the CNC test, which consists of 10 lists. Each list includes 50 monosyllabic words. Each participant was seated at the center of the double-walled audiometric booth, meeting ANSI standards (ANSI S3.1e1999). Participants were asked to listen to the word through circumaural headphones (Sennheiser HD 280; Sennheiser, Wedemark, Hanover, Germany). Participants were instructed to type what they heard on the LCD monitor in front of them. If they were unsure of the word, they took their best guess or typed ellipses to indicate that they did not know. A practice test was performed for each participant before the actual test. The practice test consisted of three separate words that the participant could see on the LCD monitor after entering what they heard. The actual test included 250 words from five randomized lists. The CNC word lists were prerecorded by one male talker. These words were presented at 65 dB SPL in the presence of two-talker babble at five signal-to-noise ratios (SNRs) (−9, −6, −3, 0, and +3 dB); one list was administered per condition. Lists number 2, 3, 4, 7, and 10 were used. Responses were scored based on the entire word (% correct; CNC-Word) and the number of phonemes (% correct; CNC-Phoneme) repeated correctly. The AzBio test assesses sentence recognition in quiet and in noise. The AzBio test was also administered in the same acoustic environment as the CNC test, and a similar customized MATLAB program was used for conducting the AzBio test. The AzBio test consists of 33 different sentence lists. Each list has 20 different sentences. Participants were asked to listen to the word through circumaural headphones (Sennheiser HD280). A practice test was administered before the actual test. The practice test had three sentences wherein the participant could look at the actual sentence after typing in what they heard. The actual test consisted of 100 sentences from 5 randomized lists spoken by two male and two female talkers in a randomized manner. These sentences were presented at 65 dB SPL in the presence of two-talker babble at five SNRs (−9, −6, −3, 0, and +3 dB); one list was administered per condition. Lists number 2, 3, 4, 5, and 10 were used. Responses were scored based on the percentage of words repeated correctly for sentences at different SNRs. After the AzBio test, the session I was terminated. An appointment for the second session was scheduled at the lab. Both testing sessions occurred within one or two weeks of each other. Participants were also instructed to avoid loud sound exposure at least 12 h before their second testing session.

### 2.4. Second Session

The second session started with the DPOAE test for evaluating the outer hair cell functioning of the cochlea (inner ear). DPOAEs of all participants were measured using a commercial system (Smart DPOAE—Intelligent Hearing Systems, Miami, FL, USA) connected to an ER- 10 D probe (Etymotic Research. Inc., Elk Grove Village, IL, USA) across the range of frequencies from 500 Hz to 6 kHz. DPOAEs at 2F1-F2 were obtained for F2 values ranging from 500 to 6000 Hz in two data points per octave. A stimulus-level combination of 65/55, sound pressure level (SPL), and stimulus frequency ratio of 1.22 were used. The DPOAE test was followed by the ABR test. The ABR test was conducted using a commercial system (Duet, Intelligent Hearing Systems, Miami, FL, USA) in the same environment as in session I. ABRs were obtained using a one-channel electrode montage with a mastoid-placed electrode from the left ear. The stimulus and acquisition parameters set to record ABRs are shown in Table 1. The left ear was selected for ABR because the noise-induced damage is more prevalent in the left ear than in the right ear [28,66,67].The non-inverting and ground electrodes were placed on the participant’s forehead (Fz) and low forehead (Fpz), while the inverting electrode was placed on the mastoid of the left ear. These areas were prepped using alcohol wipes and a Nuprep skin prep gel to effectively reduce the inter-electrode impedance values. Impedance values at each electrode site were monitored to remain below 3 kOhms with an inter-impedance value of less than 2 kOhms. These impedance values were monitored throughout the testing procedure. ABR stimuli were presented with alternating polarity at rates 11.3, 51.3, and 81.3/using insert earphones (ER-3A, St. Paul, MN, USA). ABR responses were obtained using 100 ms click stimuli presented at 80 dB nHL (85.7 ± 0.3 dB SPL, calibration in an IEC-711ear simulator). At each stimulus rate, two replications of 2000 sweeps were collected for analysis. Recording parameters included a gain of 100,000 and band-pass filtering from 100 Hz to 3000 Hz. The artifact rejection threshold was set at 31 mV. ABRs were collected with a pre-stimulus window of 12.5 ms, a post-stimulus window of 12.5 ms, and a sampling frequency of 40,000 Hz.

The ABR test was followed by the P300 test. P300 testing was done with the same system (Duet, Intelligent Hearing Systems, Miami, FL, USA). A two-channel montage was used, with channel A assigned for P300 measures and channel B for measuring and monitoring eye movements and eye blink artifacts. The stimulus and acquisition parameters set to record P300 are shown in Table 1. The prerecorded speech tokens stimuli used were the consonant–vowel /ba/as frequent stimuli (80%) and/ta/as infrequent stimuli (20%). The stimuli sequence was presented monoaurally to the left ear of each participant at 80 dB SPL. In total, 500 stimuli were used (100 rare and 400 frequent) to obtain the P300 responses.

### 2.5. Electrophysiological Waveform Analysis

After completing the ABR test, the two replications at each rate were averaged, and the averaged waveforms were utilized for evaluating the ABR I and V waveforms. The amplitude of ABR waves I and waves V was calculated from the voltage difference between the identified positive peak and the following trough. Similarly, the P300 amplitude was calculated from the voltage difference between the identified positive peak and the following trough. Two audiologists identified the waveforms separately. Any disagreement pertaining to the peak measurements between two audiologists was resolved by them reviewing the data together.

### 2.6. Statistical Analysis

Both descriptive and inferential statistical analyses for this study were performed using IBM SPSS (version 26.0; IBM Corp.: Armonk, NY, USA). Multivariable linear regression modeling was used to estimate the relationship between NEB and peripheral auditory electrophysiological measures (wave I and V amplitude). To estimate the influence of NEB on ABR wave I and V amplitudes while controlling the effect of gender, the ABR wave I and V amplitudes were included as continuous dependent variables, and gender and NEB as independent variables. Similar analyses were performed to estimate the relationship between NEB and central auditory electrophysiological measures (P300 amplitude and latency). The relationship between NEB and speech-in-noise measures at the word level (CNC) and sentence level (AzBio test) at five SNRs was also estimated using linear regression modeling.

Mixed effect linear regression models were used to study the effects of non-musician/musician groups, gender, and SNR and interaction between these factors on CNC and AzBio measures. The subjects were considered a random variable in these analyses.

## 3. Results

### 3.1. Descriptive Statistics

**Figure 1:**
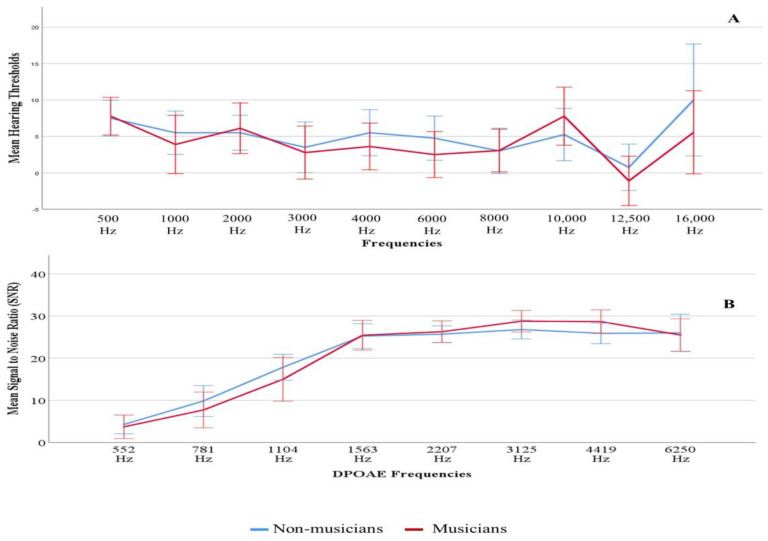
Mean hearing thresholds (Panel (**A**)) and mean DPOAE SNRs (Panel (**B**)) of left ear with standard error according to groups.

**Figure 2:**
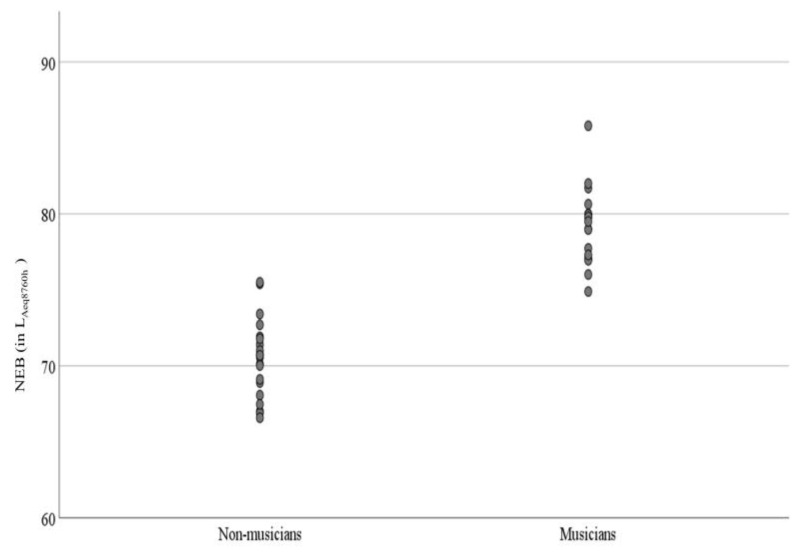
Simple scatter plot of NEB as a function of groups.

A total of 38 participants (17 females, 21 males) from 18–30 years were included in this study (mean age 21.9 years). These 38 participants were divided into two groups, non-musicians (10 females, 10 males) and musicians (7 females, 11 males). The group means audiograms of musicians and non-musicians are shown in Figure 1 (Panel A). As specified by the inclusion criteria, both musician and non-musician groups had thresholds within clinically normal limits (≤15 dB HL) for the octave frequencies 500 to 8000 Hz. There was increased variability at the extended high frequencies, particularly at 16 kHz. However, there were no significant group differences at any frequencies from 500 to 16 kHz (Supplementary Material S2). The average hearing thresholds at 3, 4, 6 kHz (PTA346) were calculated because the effect of noise exposure is higher on these frequencies [66]. Likewise, the average of EHF hearing thresholds (PTA 101216) was also calculated. PTA346 and PTA101216 were not statistically different between non-musicians and musician groups (See Supplementary Material S2). Outer hair cell functioning was evaluated by recording DPOAEs at frequencies 0.5 to 6 kHz. DPOAE amplitudes (Figure 1, panel B) were not statistically significant between the two groups (see Supplementary Material S2). An independent sample *t*-test revealed that the mean NEB between non-musicians and musicians groups was significantly different. The mean NEB was higher for musicians compared to the mean for non-musicians (mean (musician)-mean (non-musician) = 8.65 *L_A_*_eq8760h_, *p* < 0.001). The mean NEB of non-musicians and musician groups was 70.46 and 79.11 *L_A_*_eq8760h_, respectively. Figure 2 shows NEB data as a function of experimental and control groups. The mean differences in NEB between non-musician and musician groups were attributed to our sampling scheme. The results of the linear regression revealed no statistically significant linear association between NEB and PTA 101216 (r(36) = −0.068, *p* = 0.680). Similar analysis showed no linear association between NEB and PTA 346 kHz (r(36) = −0.045, *p* = 0.780).

### 3.2. Electrophysiological Measures

**Figure 3:**
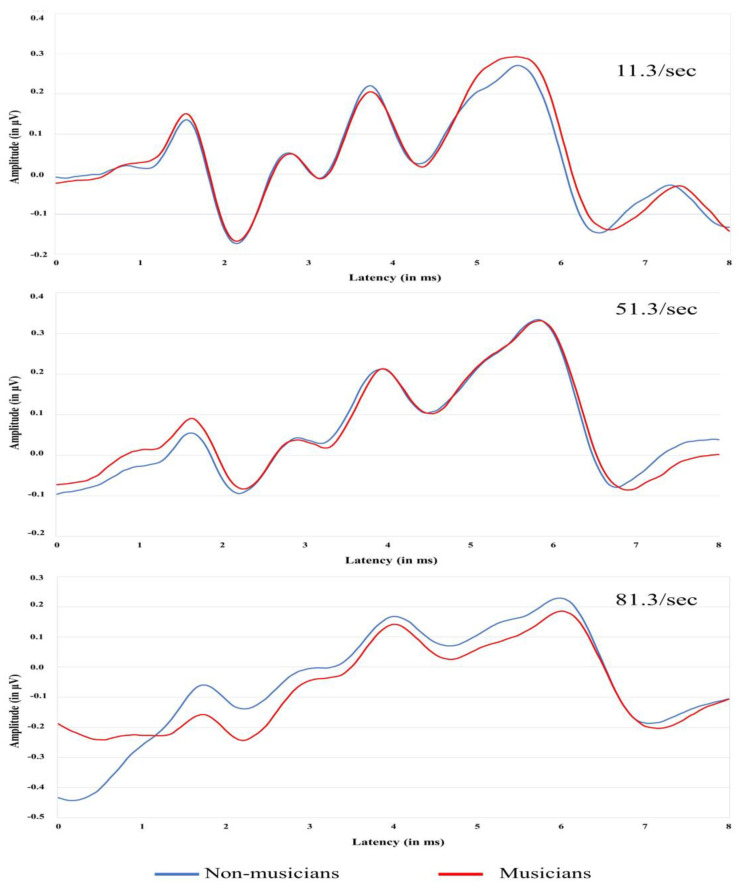
Grand average ABR waveforms of two groups collected at three stimulus repetition rates 11.3, 51.3, and 81.3/s. The *X*-axis corresponds to latency, and the *Y*-axis corresponds to ABR wave amplitude measured in µV.

**Figure 4:**
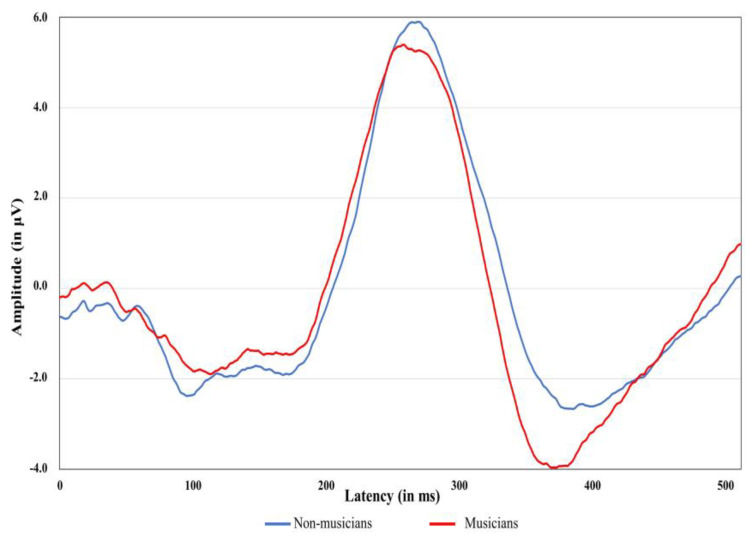
Grand average P300 waveforms of two groups. The *X*-axis corresponds to latency, and the *Y*-axis corresponds to ABR wave amplitude measured in nV.

Table 2 shows means and standard deviations for latencies and amplitude of ABR waves I and V obtained at rates of 11.3, 51.3, and 81.3 clicks per second according to gender. The means and standard deviations for latencies and amplitudes of P300 of 34 participants [18 non-musicians (9 females, 9 males); 16 musicians (6 females, 10 males)] are shown in Table 3. The P300 data of four participants were not included due to poor wave morphology and artifacts. The grand average ABR and P300 waveforms of musicians and non-musicians are shown in Figure 3 and Figure 4, respectively. The wave I amplitude is highest at rate 11.3/s, and decreases at higher stimulus rates. The results of the regression analyses for examining the relationship between NEB and ABR measures are shown in Table 4. The relationship between the NEB and the amplitude of wave I and between the NEB and wave V at three stimulus rates was investigated while controlling the effects of gender. The NEB revealed no significant association with wave I and wave V amplitudes at all three stimulus rates (See Figure S1 in Supplementary Material S3). Similar regression analyses were also performed to investigate the relationship between the group and ABR measures. The group revealed no significant association with wave I and wave V’s amplitude at all three stimulus rates.

Regression analyses were also performed to study the relationship between NEB and P300 amplitude and latency while controlling the effect of gender. There was no significant association between NEB and P300 amplitude (*R*^2^ = −0.043, *F*(2,31) = 0.315, *p* = 0.732), and latency measure (*R*^2^ = 0.034, *F*(2,31) =1.579, *p* = 0.222), (See Figure S2 in Supplementary Material S3. The relationship between groups and P300 amplitude and latency were examined using regression analyses while controlling the effect of gender. There was no significant association between groups and P300 amplitude (*R*^2^ = −0.052, *F*(2,31) = 0.184, *p* = 0.833) and latency measure (*R*^2^ = 0.015, *F*(2,31) =1.250, *p* = 0.300).

### 3.3. Word Recognition in Noise

**Figure 5:**
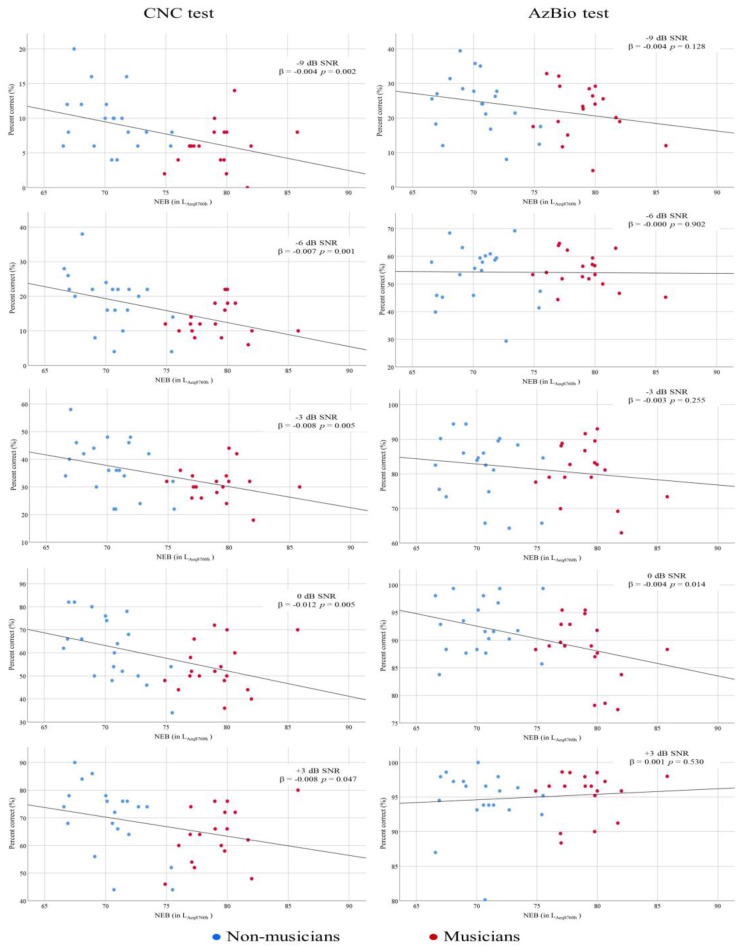
Scatter plots for NEB and CNC test performance (**left** panel) and NEB and AzBio test performance (**right** panel). Linear regression lines were inserted to show the predictive relationship. The signal to noise ratio (SNRs) is denoted in each panel (−9, −6, −3, 0, and +3 dB SNR). Regression results are shown in each panel.

Word recognition in noise was examined in all the participants using the CNC test at +3, 0, −3, −6, and −9 dB SNRs. The relationship between NEB and the performance on CNC test at different SNRs was investigated while controlling the confounding effect of gender. NEB showed a significant association with performance on CNC measures at all SNRs. Table 5 shows the results of regression analyses of NEB and CNC measures. The adjusted R^2^ values for the models ranged from 0.079 to 0.245, suggesting that a small portion of the variance in the dependent variables was exclusively attributed to NEB. Figure 5 (left panel) reveals a significant negative relationship between NEB and performance in the CNC test at all five SNRs. In addition, the effect of groups, gender, SNRs and interaction between these variables on CNC measures were evaluated using mixed model linear regression. The results of this analysis are shown in Supplementary Material S4. The main effects of groups (*F*(1,34) = 8.630; *p* = 0.006) and SNRs (*F*(4,140) = 526.737; *p* = 0.000) were statistically significant, while the main effect of gender (*F*(1,34) = 1.623; *p* = 0.211) was not statistically significant. Furthermore, there was not strong evidence of interaction between groups and gender (*F*(1,34) = 0.772; *p* = 0.386), gender and SNRs (*F*(4,140) = 0.210; *p* = 0.932), and between groups and SNRs (*F*(4,140) = 0.684; *p* = 0.604). This finding shows that the overall performance of non-musicians on the CNC test was significantly better than that of musicians.

### 3.4. Sentence Recognition in Noise

Table 6 shows the results of regression analyses for examining the relationship between NEB and performance on the AzBio test at +3, 0, −3, −6, and −9 dB SNRs. The relationship between NEB and the performance on the AzBio test at different SNRs was investigated while controlling the confounding effect of gender. NEB showed a significant association with performance on the AzBio test at 0 dB SNR. At all other four SNR conditions, there was no significant association between NEB and performances on the AzBio test. Figure 5 (right panel) displays the scatter plots between the NEB and performance on the AzBio test of non-musicians and musicians at +3, 0, −3, −6, and −9 dB SNRs.

The results of a mixed model linear regression analysis examining the effects of group, gender, SNRs and interaction between these variables on AzBio measures are shown in Supplementary Material S4. The main effect of SNRs was significant (*F*(4,140) = 1002.669; *p* = 0.000). The main effect of groups (*F*(1,34) = 0.302; *p* = 0.586) and gender (*F*(1,34) = 0.082; *p* = 0.776) were not statistically significant. Similarly, the interaction between groups and gender (*F*(1,34) = 0.310; *p* = 0.581), gender and SNRs (*F*(4,140) = 0.763; *p* = 0.551) and between groups and SNRs (*F*(4,140) = 1.268; *p* = 0.285) were also not statistically significant. The results of this analysis indicates that the performance of non-musicians in the AzBio test was not significantly different from that of musicians.

## 4. Discussion

The present study aimed to investigate the effect of noise exposure history on the peripheral and central auditory system, and on performance on speech-in-noise tests. It was hypothesized that the influence of high noise exposure on peripheral and central auditory systems would be manifested in the form of compromised electrophysiological and speech-in-noise measures in normal-hearing collegiate students with high NEB. We obtained supporting evidence for this hypothesis, suggesting that musicians with high NEB exhibit poorer speech-in-noise performance than their non-musicians counterparts.

### 4.1. The Relationship between NEB and Performances on Speech-in-Noise Tasks

We recruited musicians with high NEB and non-musicians with low NEB. We obtained a significant main effect for groups in the CNC test and a negative relationship between NEB and CNC scores at –9, −6, −3, 0, and +3 dB SNRs. As shown in Figure 5, the relationship between NEB and performance on the CNC test is consistent at all five SNRs, indicating that high NEB might compromise suprathreshold speech perception abilities among young musicians. Similarly, the result of mixed linear regression indicates that musicians perform poorer compared to non-musicians. The difference between the groups does not reach the conventional *p* < 0.05 level of statistical significance at any SNR, which could possibly be due to the smaller sample size of our study. Further research with a larger sample size is warranted to clarify more definitively the implications of these findings.

We obtained no association between NEB and sentence recognition in noise performance in the AzBio test at –9, −6, −3, and +3 dB SNRs. Similarly, the main effect for groups in the mixed model linear regression analysis was also not statistically significant. We found a significant negative relationship between NEB and AzBio test performance at 0 dB SNR, as shown in Figure 5. The discrepancy between the findings of the CNC and AzBio tests could be attributed to the stimuli used in these two tests. Cognitive and linguistic factors might influence the performance on AzBio tests, but they might exhibit a lower influence on CNC scores. Although a sentence may be a realistic stimulus with better face validity, the contextual cues contribute heavily to intelligibility and make basic auditory functions difficult to determine [68]. A few studies have reported an approximate difference of 6–7 dB SNR in the speech recognition performance of words and sentences among adults, with sentences always requiring lower SNR than words [69,70].

The observed negative trend between NEB and word recognition in noise performance on the CNC test might be influenced by the effect of noise exposure on central auditory structures. The results of the CNC tests are consistent with the findings of previous studies on normal-hearing adults with high noise exposure histories [43,44,51]. Some studies on adults with high noise exposure have found no association between speech-in-noise performance and noise exposure history [42,54,71,72]. Further research is required to quantify the influence of cochlear synaptopathy on suprathreshold speech perceptions.

### 4.2. The Relationship between NEB and Electrophysiological Measures

The findings of the present study showed no relationship between NEB and ABR wave I amplitude obtained at low (11.3/s), medium (51.3/s), and high (81.3/s) stimulus repetition rates. We could not find any difference between ABR wave I between musicians and non-musicians. As is apparent in Figure 3, there is no significant association between NEB and ABR wave I amplitude and between NEB and ABR wave V amplitude. Our past study indicated a modest association between NEB and ABR wave I amplitude in young musicians and non-musicians [65]. The present study could not replicate these findings, possibly due to our smaller sample size and the high inter-subject variability in audiological measures. We observed that the standard error (*SE*) of a mean for ABR wave I amplitude obtained at rate 11.3 (SE_11.3wave I_ = 0.019 µV) was higher than the *SE* of a mean for ABR wave I obtained at rate 51.3 (SE_51.3wave I_ = 0.013 µV) and 81.3 (SE_81.3wave I_ = 0.012 µV). A similar trend was observed for ABR wave V amplitude (SE_11.3wave I_ = 0.021 µV, SE_51.3wave I_ = 0.19 µV, SE_81.3wave I_ = 0.020 µV). These findings correspond with the results of other studies investigating the association between noise exposure and electrophysiological measures [49,50,51,54,73]. This result is in accordance with some previous studies on different study populations [49,53,54,74]. The first possible explanation for this insignificant finding could be the higher variability of auditory evoked potentials, particularly ABR wave I in humans. In a study by Prendergast et al. [50], the coefficient of variation for ABR waves I amplitude was 25% in the low noise exposure group, and this may indicate a substantial degree of variability compared to the effect being measured. Washnik et al. [65] also reported higher variability in ABR wave I amplitude obtained at 90, 75, and 60 dB nHL. The differences in adult head size and geometry might also contribute to the inter-subject variability and reduced statistical power to identify differences in auditory electrophysiological measures in the human population [75,76]. In addition, there is another possibility that noise exposure induces cochlear synaptopathy only in selected portions of the cochlea [29,30,38], and therefore, the effect of cochlear synaptopathy is enshrouded when ABRs are evoked by transient click stimuli, which present energy in a broad frequency range.

Furthermore, no significant influence of NEB on P300 amplitude and latency was found in the present study. The P300 measures are reflective of attentional capacity. Many studies have reported that musical training enhances neural coding to discriminate subtle differences, leading to enhanced discrimination abilities of the brain; this is manifested in the form of shorter P300 latencies and higher P300 amplitude among musicians when compared to non-musicians [77,78]. Our P300 amplitude and latency findings are consistent with other studies on the human population with high noise exposure history [79,80]. Thakur and Banerjee [79] studied the influence of high noise exposure on the central auditory pathway using P300 among ground crew members of an airport. They found no significant difference in P300 amplitude and latency between the experimental and control groups. One reason for the lack of significant association between NEB and P300 could be the sample size. Future research is needed to investigate the influence of noise exposure on auditory–cognitive responses such as P300.

### 4.3. Speech-in-Noise and Electrophysiological Measures in Musicians

Several studies have shown musicians’ advantage in speech-in-noise (SIN) perception [4,14,81,82]. In contrast, others reported no significant difference in SIN performances between musicians and non-musicians [13,83,84,85]. A possible factor influencing these mixed findings is inter-subject variability in noise exposure among musicians. Musicians are regularly exposed to high sound levels during large and small ensemble rehearsals, individual practice sessions, music performances, and listening to music pieces through speakers or headphones. Skoe et al. [86] found that noise exposure among musicians suppresses the musicians’ SIN perception advantage. The result of our study indicates that noise exposure is negatively associated with SIN performance at the word level among musicians. Though our speech-in-noise findings are in line with the investigation by Liberman et al. [43] and Hope et al. [87], other researchers found no significant relationship between noise exposure history and SIN performance [48,49,53,72]. The null results of the SIN measures in the above studies could be related to methodological factors, such as the complexity of stimuli and their difficulty levels. Valderrama et al. [48] and Yeend et al. [72] used sentences in the listening in spatialized noise—sentences high cue condition (LiSN-S) test, which may be influenced by cognitive factors. Other studies on humans with history of high noise exposure utilized SIN measures such as word-in noise (WIN) tests [49,53]; however, these studies administered WIN tests at SNRs ranging from 0 to 30 dB, which was comparatively higher than the SNRs used for CNC test in our study. Le Prell [88] suggested that studies incorporating the most difficult SIN tasks may show greater sensitivity to the detection of the relationship between noise exposure and SIN performance. In a recent systematic review, DiNino et al. [89] mentioned that speech-in-noise tests that use low SNRs and maximize minute sensory details by using stimuli that offer minimal lexical, syntactic, or semantic cues are more likely to show an interest in the relationship between human studies and HHL.

Speech-in-noise measures, particularly the CNC test in our study, have shown that NEB is negatively related to speech-in-noise performance, and that the overall performance of musicians as a group is significantly poorer than non-musicians. On the other hand, the outcomes of the electrophysiological measures, such as amplitude and latency of P300 and ABR wave I and V, showed no association with NEB. The insignificant findings in the ABR measures of this study could also be associated with the possibility that noise exposure induces synaptopathy only in certain regions of the cochlea [29,30,38]; hence, the effect of synaptopathy becomes obscured when ABR are evoked by broad-range frequency stimuli such as clicks.

The results of the current study show that despite similar peripheral processing (DPOAE responses and ABR wave I amplitude), speech-in-noise performance with CNC words was reduced in individuals with high NEB. Recent investigations have revealed the negative influence of noise exposure on human cognition [90,91,92,93]. Patel et al. (2022) suggested that high-level cognitive tasks and their corresponding brain regions are not equally susceptible to high noise exposure [93]. Thus, it can be hypothesized that the central processing (except the central processes involved in P300 generation) involved in understanding speech in highly demanding situations, such as CNC words in noise, might be more prone to the negative effects of noise exposure, and this might be manifested in the form of reduced performance in speech-in-noise tasks at the word level. Unfortunately, the current study did not measure specific cognitive domains such as working memory and other executive functions contributing to speech-in-noise performance. The specific central processes involved in the reduced speech-in-noise performance of individuals with high NEB need to be further explored in future studies.

### 4.4. Study Limitations and Future Directions

There are a few methodological factors that may have influenced the outcomes of this study, and these should be considered when interpreting the results. Firstly, noise exposure was measured using a retrospective noise exposure questionnaire developed by Johnson et al. [61], and this questionnaire estimates the amount of noise exposure in one year. Although many studies have used this questionnaire [49,51,53,73], it does not include a comprehensive list of noise exposure areas and does not account for noise exposure beyond an individual’s last 12 months of noise exposure. On the other hand, other studies measured noise exposure across a lifetime [40,48,50], and performed noise dosimetry measurements [45,86] to obtain real and more accurate noise exposure data. As the effect of noise exposure is cumulative, an estimate of noise exposure will be more accurate if noise dosimetry measurements are performed along with the administration of a lifetime noise exposure questionnaire. Future studies should include noise dosimetry measurements and a lifetime noise exposure questionnaire to obtain more reliable and accurate noise exposure data which can be compared with auditory electrophysiological and behavioral measures.

In addition, there is no widely accepted standard protocol for evaluating cochlear synaptopathy in the human population, and it can be argued that the evoked potential metrics other than the one used by the present study might be more sensitive in detecting cochlear synaptopathy in humans [94]. The inclusion of non-musicians and musicians with different ranges of noise exposure is another critical factor. All the student musician participants had *L_A_*_eq8760h_ values higher than 76, while all the non-musicians had *L_A_*_eq8760h_ values below 76. Such division in the noise exposure range between musicians and non-musicians may influence the findings of this study.

Cognitive factors such as working memory and non-verbal IQ that have been linked to speech recognition in noise abilities were not measured in the current study. Previous investigations have revealed that greater WM capacity is associated with enhanced speech-in-noise perception abilities [4,84,95] Recent findings indicate that the cognitive abilities of the individual may be the crucial factor in their speech recognition in noise ability, rather than musicianship [83,84]. Hence, the reduced performance on the CNC test among student musicians cannot be solely attributed to noise exposure, because the effect of non-verbal IQ and WM cannot be ruled out.

With regard to the set of tests utilized in the current study, there are a few other sensitive auditory tests, such as the threshold equalizing nose (TEN) test and contralateral OAE suppression, whose inclusion into the test battery might have provided better insight into the association between noise exposure and speech-in-noise deficits. Lastly, the participants in the current study were of European ethnicity. Thus, the results of this study should not be generalized beyond individuals of European ethnicity. Future studies can address the influence of noise exposure on auditory electrophysiological and perceptual measures among other ethnic groups.

## 5. Conclusions

The findings of several studies investigating noise-induced HHL in humans have been mixed [40,41,42,43,49,50,51,52,53,54,72]. The present study obtained a significant association between noise exposure and word-level speech-in-noise measures. However, we did not find any association between NEB and any electrophysiological measures used in the present study. These findings indicate that noise exposure may affect the central auditory structures. We found a negative relationship between NEB and speech recognition in noise at the word level. More interestingly, we found that musicians perform more poorly than non-musicians on word-level tasks, but not on sentence-level tasks. Collectively, these results suggest that musicians with high NEB could lose their perceptual advantage for processing words in background noise over non-musicians. The null findings in the AzBio test suggest that the deficit in speech processing at the word level was compensated at the sentence level. These results might indicate that musicians with high NEB exhibit a cognitive advantage which influences speech processing at the sentence level [96]. Future research is needed to test the influence of high noise exposure on auditory–cognitive measures in musicians and non-musicians.

## Figures and Tables

**Table 1:** Stimulus and acquisition parameters set to record ABR and P300.

P300 Stimulus Parameters		ABR Stimulus Parameters
Stimulus	/ba/—frequent (80%) /ta/—infrequent (20%)	100 µs click
Intensity	80 dB nHL	80 dB nHL
Stimulation rate	1.10/s	11.3, 51.3, and 81.3/s
Transducer	ER-3A insert earphones	ER-3A insert earphones
Presentation	Monoaural (left ear)	Monoaural (left ear)
**Recording Parameters**		
Filter setting	1–30 Hz	100–3000 Hz
Electrode montage	Vertical (2-channel) Channel A: Positive Cz Negative mastoid left Channel B: Positive above left eye Negative below left eye Ground: Fpz	Vertical (1-channel) Positive Cz Negative left mastoid Ground Fpz

**Table 2:** The average amplitude and latency of waves I and V for each stimulus rate. The standard deviations are listed in parentheses.

Stimulus Rate	Group	Gender	Wave I Amplitude	Wave V Amplitude	Wave I Latency	Wave V Latency
			Mean (SD)	Mean (SD)	Mean (SD)	Mean (SD)
11.3	Non-musician	Male	0.32 (0.14)	0.46 (0.14)	1.51 (0.11)	5.72 (0.17)
		Female	0.35 (0.11)	0.54 (0.11)	1.59 (0.09)	5.64 (0.22)
	Musician	Male	0.30 (0.10)	0.44 (0.12)	1.58 (0.10)	5.72 (0.17)
		Female	0.38 (0.13)	0.61 (0.12)	1.59 (0.03)	5.59 (0.25)
51.3	Non-musician	Male	0.15 (0.08)	0.41 (0.14)	1.63 (0.13)	5.92 (0.17)
		Female	0.18 (0.10)	0.53 (0.06)	1.66 (0.10)	5.92 (0.19)
	Musician	Male	0.19 (0.09)	0.43 (0.11)	1.66 (0.12)	5.99 (0.23)
		Female	0.21 (0.04)	0.49 (0.13)	1.66 (0.08)	5.88 (0.18)
81.3	Non-musician	Male	0.12 (0.10)	0.40 (0.14)	1.71 (0.10)	6.10 (0.13)
		Female	0.15 (0.08)	0.54 (0.09)	1.70 (0.11)	6.11 (0.19)
	Musician	Male	0.11 (0.07)	0.38 (0.11)	1.68 (0.15)	6.17 (0.18)
		Female	0.17 (0.04)	0.44 (0.10)	1.72 (0.07)	6.05 (0.14)

**Table 3:** The average amplitude and latency of P300. The standard deviations are listed in parentheses.

Group	Gender	P300 Amplitude	P300 Latency
		Mean (SD)	Mean (SD)
Non-musician	Male	9.22 (4.54)	274.78 (14.70)
	Female	11.84 (6.03)	263.89 (23.31)
Musician	Male	11.38 (3.33)	276.70 (15.08)
	Female	10.33 (6.17)	267.83 (27.39)

**Table 4:** Results of the regression analyses listing predictors for ABR wave I and V amplitudes (μV).

		Wave I Rate 11.3	Wave I Rate 51.3	Wave I Rate 81.3	Wave V Rate 11.3	Wave V Rate 51.3	Wave V Rate 81.3
NEB	β value	0.003	0.004	0.001	0.005	0.000	−0.005
	Std. error	0.004	0.003	0.002	0.004	0.004	0.004
	*p*-value	0.417	0.132	0.670	0.262	0.990	0.220
Gender	β value	0.064	0.025	0.045	0.129	0.110	0.100
	Std. error	0.038	0.027	0.025	0.040	0.037	0.038
	*p*-value	0.106	0.355	0.077	0.003	0.006	0.012
	Adjusted R^2^	0.028	0.023	0.035	0.236	0.156	0.174
	*p*-value	0.229	0.251	0.203	0.009	0.020	0.013

**Table 5:** Results of the regression analyses listing predictors for performances in the CNC test at 5 SNRs.

CNC Test		−9 dB	−6 dB	−3 dB	0 dB	+3 dB
NEB	β value	−0.004 **	−0.007 **	−0.008 **	−0.012 **	−0.008 *
	Std. error	0.001	0.002	0.003	0.004	0.004
	*p*-value	0.002	0.001	0.005	0.005	0.047
Gender	β value	−0.025 *	−0.033	−0.019	−0.033	−0.047
	Std. error	0.012	0.021	0.027	0.039	0.037
	*p*-value	0.040	0.117	0.477	0.398	0.218
	Adjusted R^2^	0.237 **	0.245 **	0.160 *	0.158 *	0.079 *
	*p*-value	0.003	0.003	0.018	0.019	0.090

* *p* < 0.05, ** *p* < 0.01; unstandardized coefficients b and adjusted R^2^ values are listed.

**Table 6:** Results of the regression analyses listing predictors for performances in the AzBio test at five SNRs.

AzBio Test		−9 dB	−6 dB	−3 dB	0 dB	+3 dB
NEB	β value	−0.004	0.000	−0.003	−0.004 *	0.001
	Std. error	0.003	0.003	0.003	0.002	0.001
	*p*-value	0.128	0.902	0.255	0.014	0.530
Gender	β value	0.027	−0.006	−0.018	0.004	0.002
	Std. error	0.025	0.028	0.029	0.017	0.013
	*p*-value	0.304	0.841	0.537	0.833	0.885
	Adjusted R^2^	0.054	−0.056	−0.013	0.120 *	−0.045
	*p*-value	0.143	0.975	0.470	0.040	0.818

* *p* < 0.05, unstandardized coefficients b and adjusted R^2^ values are listed.

## Data Availability

The data presented in this study are not publicly available due to ethical constraints, but are available on request from the corresponding author.

## Supplementary Materials

The following supporting information can be downloaded at: https://www.mdpi.com/article/10.3390/diagnostics13050934/s1, Supplementary Material S1: Noise exposure questionnaire for estimating the noise exposure in the last 12 months. This questionnaire was developed by Johnson et al. [61]; Supplementary Material S2: Tables of independent *t*-test for pure-tone hearing thresholds and DPOAE SNR across frequencies in left ear; Supplementary Material S3: Figure S1. Scatter plots for NEB and ABR measures. Figure S2. Scatter plots for NEB and ABR measures; Supplementary Material S4: Tables of mixed model linear regression analysis for CNC and AzBio tests.
