# Supplementary material for: Auditory Electrophysiological and Perceptual Measures in Student Musicians with High Sound Exposure
Source: Diagnostics (Basel). 2023 Mar 1;13(5):934. doi: 10.3390/diagnostics13050934 (PMC10000734; doi:10.3390/diagnostics13050934)
Supplement: Supplementary file 1 [file diagnostics-13-00934-s001.zip › diagnostics-2013269-supplementary.pdf]

Supplementary Material S1

Noise Exposure Questionnaire

INSTRUCTIONS:

- ***Please answer the following questions about yourself, your hearing, and any noise you may have been around during the past year. Write an answer in the blank [______] or check [√] the best answer to each question.***
- ***Be sure to answer all the questions.***

Please answer these general questions about your hearing and any loud sounds. **DURING THE PAST YEAR (12 months):**	
1.	How often were you around or did you shoot firearms such as rifles, pistols, shotguns, etc.? ☐ Never ☐ Every few months ☐ Monthly ☐ Weekly ☐ Daily
2.	How often were you exposed to loud sounds while working on a paid job? By loud sounds, we mean sounds so loud that you had to shout or speak in a raised voice to be heard at arm's length. ☐ Never ☐ Every few months ☐ Monthly ☐ Weekly ☐ Daily
3.	How often were you exposed to any other types of loud sounds, such as power tools, lawn equipment, or loud music? By loud sounds, we mean sounds so loud that you had to shout or speak in a raised voice to be heard at arm's length. ☐ Never ☐ Every few months ☐ Monthly ☐ Weekly ☐ Daily
4.	How often were you exposed to loud sound that made your ears "ring" or "buzz"? ☐ Never ☐ Every few months ☐ Monthly ☐ Weekly ☐ Daily
5.	How often were you exposed to loud sound that made your hearing seem muffled for awhile? ☐ Never ☐ Every few months ☐ Monthly ☐ Weekly ☐ Daily
6.	How often were you exposed to loud sound that made your ears hurt, feel "full," or bother you in any other way? ☐ Never ☐ Every few months ☐ Monthly ☐ Weekly ☐ Daily
Please answer these detailed questions about any loud sounds. **DURING THE PAST YEAR (12 months):**	
7.	Outside of a paid job, how often did you use power tools, chainsaws, or other shop tools? ☐ Never ☐ Every few months ☐ Monthly ☐ Weekly ☐ Daily<br>If you used power tools, on average, how many hours did each time/session last? ☐ 8 hours or more ☐ 4 hours up to 8 hours ☐ 1 hour up to 4 hours ☐ Less than 1 hour<br>If you used power tools, how often did you wear earplugs or earmuffs during this activity? ☐ Never ☐ Sometimes ☐ Always
8.	Outside of a paid job, how often did you drive heavy equipment or use loud machinery (such as tractors, trucks, or farming or lawn equipment like mowers/leaf blowers)? ☐ Never ☐ Every few months ☐ Monthly ☐ Weekly ☐ Daily<br>If you drove/used loud machinery, on average, how many hours did each time/session last?

☐ 8 hours or more ☐ 4 hours up to 8 hours ☐ 1 hour up to 4 hours ☐ Less than 1 hour

If you drove/used machinery, how often did you wear earplugs or earmuffs during this activity?

☐ Never ☐ Sometimes ☐ Always

9. How often did you attend car/truck races, commercial/high school sporting events, music concerts/dances or any other events with amplified public announcement (PA)/music systems?

☐ Never ☐ Every few months ☐ Monthly ☐ Weekly ☐ Daily

If you attended these events, on average, how many hours did each time/session last?

☐ 8 hours or more ☐ 4 hours up to 8 hours ☐ 1 hour up to 4 hours ☐ Less than 1 hour

If you attended these events, how often did you wear earplugs or earmuffs during this activity?

☐ Never ☐ Sometimes ☐ Always

10. How often did you ride/operate motorized vehicles such as motorcycles, jet skis, speed boats, snowmobiles, or four-wheelers?

☐ Never ☐ Every few months ☐ Monthly ☐ Weekly ☐ Daily

If you rode motorized vehicles, on average, how many hours did each time/session last?

☐ 8 hours or more ☐ 4 hours up to 8 hours ☐ 1 hour up to 4 hours ☐ Less than 1 hour

If you rode motorized vehicles, how often did you wear earplugs or earmuffs during this activity?

☐ Never ☐ Sometimes ☐ Always

11. How often did you ride in or pilot small aircraft/private airplanes?

☐ Never ☐ Every few months ☐ Monthly ☐ Weekly ☐ Daily

If you flew airplanes, on average, how many hours did each time/session last?

☐ 8 hours or more ☐ 4 hours up to 8 hours ☐ 1 hour up to 4 hours ☐ Less than 1 hour

If you flew airplanes, how often did you wear earplugs or earmuffs during this activity?

☐ Never ☐ Sometimes ☐ Always

Please continue answering these <u>detailed</u> questions about any loud sounds.

DURING THE PAST YEAR (12 months):

12. How often were you around or did you shoot firearms such as rifles, pistols, shotguns, etc.?

☐ Never ☐ Every few months ☐ Monthly ☐ Weekly ☐ Daily

If you were around/shot firearms, on average, how many shots did you fire each time/session?

__________ **shotgun/rifle** shots per session __________ **pistol** shots per session

If you were around/shot firearms, how often did you wear earplugs or earmuffs while shooting?

☐ Never ☐ Sometimes ☐ Always

13. How often did you play a musical instrument?

☐ Never ☐ Every few months ☐ Monthly ☐ Weekly ☐ Daily

If you played, please tell us what musical instrument: ____________________________________

If you played a musical instrument, on average, how many hours did each time/session last?

☐ 8 hours or more ☐ 4 hours up to 8 hours ☐ 1 hour up to 4 hours ☐ Less than 1 hour

	If you played a musical instrument, how often did you wear earplugs or earmuffs while playing?<br>☐ Never ☐ Sometimes ☐ Always
14.	How often did you listen to music, radio programs, etc. using personal <u>headsets</u> or <u>earphones</u>?<br>☐ Never ☐ Every few months ☐ Monthly ☐ Weekly ☐ Daily<br><br>If you listened through earphones, on average, how many hours did each time/session last?<br>☐ 8 hours or more ☐ 4 hours up to 8 hours ☐ 1 hour up to 4 hours ☐ Less than 1 hour
15.	Other than music concerts and headset use (*already covered in questions 9 and 14*), how often did you listen to music, radio programs, etc. from audio speakers in a car or at home?<br>☐ Never ☐ Every few months ☐ Monthly ☐ Weekly ☐ Daily<br><br>If you listened via speakers, on average, how many hours did each time/session last?<br>☐ 8 hours or more ☐ 4 hours up to 8 hours ☐ 1 hour up to 4 hours ☐ Less than 1 hour
Please continue answering these <u>detailed</u> questions.	**NOTE DIFFERENT TIME-FRAMES:**
16.	**Now think back to this past <u>summer</u>. Over the summer months,** did you work a noisy <u>paid</u> job, such as in construction, farming, a factory, lawn service, carwash, or other indoor or outdoor job working around loud equipment or machinery? By noisy job, we mean sounds so loud that you had to shout or speak in a raised voice to be heard at arm's length.<br>☐ Yes ☐ No *(if no, skip to # 17.)*<br><br>If yes, please describe this noisy job: ____<br><br>If you worked a noisy job, please estimate the number of hours you worked in a typical week:<br><br>_________ hours worked per typical week this summer<br><br>If you worked a noisy job this summer, did your employer give you earplugs or earmuffs to wear at work?<br>☐ Yes ☐ No<br><br>How often did you wear earplugs or earmuffs when around loud noise at this summer job?<br>☐ Never ☐ Sometimes ☐ Always
17.	**<u>Other than</u> during the summer, over the past year**, did you work one or more noisy <u>paid</u> jobs, such as in construction, farming, a factory, lawn service, carwash, or other indoor or outdoor job working around loud equipment or machinery? By noisy job, we mean sounds so loud that you had to shout or speak in a raised voice to be heard at arm's length.<br>☐ Yes ☐ No *(if no, you're done with the survey)*<br><br>If yes, please describe the noisy job(s): ____<br><br>If you worked a noisy job, please estimate the number of hours you worked in a typical week:<br><br>_________ average hours worked per typical week during the school year<br><br>If you worked a noisy job during the school year, did your employer give you earplugs or earmuffs to wear at work? ☐ Yes ☐ No<br><br>How often did you wear earplugs or earmuffs when around loud noise at this noisy job(s)?<br>☐ Never ☐ Sometimes ☐ Always

Supplementary Material S2

Independent t test for pure-tone hearing thresholds across frequencies in the left ear

Hearing Thresholds	Groups	N	Mean	SD	Test statistic (*t*)	Significance (*p*)
PTA 346 kHz	Non-musicians	40	4.66	5.91	0.96	0.339
	Musicians	38	2.96	4.80		
PTA 101216 kHz	Non-musicians	40	5.33	8.26	0.48	0.630
	Musicians	38	4.07	7.67		
0.5 kHz	Non-musicians	40	7.50	5.50	-0.156	0.877
	Musicians	38	7.78	5.48		
1 kHz	Non-musicians	40	5.50	6.66	0.654	0.518
	Musicians	38	3.89	8.49		
2 kHz	Non-musicians	40	5.50	5.35	-0.294	0.770
	Musicians	38	6.11	7.38		
3 kHz	Non-musicians	40	3.50	7.79	0.287	0.776
	Musicians	38	2.78	7.71		
4 kHz	Non-musicians	40	5.50	7.05	0.837	0.408
	Musicians	38	3.61	6.81		
6 kHz	Non-musicians	40	4.75	6.78	1.027	0.311
	Musicians	38	2.50	6.69		
8 kHz	Non-musicians	40	3.00	6.95	-0.026	0.980
	Musicians	38	3.06	6.21		
10 kHz	Non-musicians	40	5.25	8.02	-0.946	0.350
	Musicians	38	7.78	8.44		
12.5 kHz	Non-musicians	40	0.75	7.12	.801	0.428
	Musicians	38	-1.11	7.18		
16 kHz	Non-musicians	40	10	17.16	0.912	0.368
	Musicians	38	5.56	12.13		

Independent t test for DPOAE SNR across frequencies in the left ear

DPOAE Amplitude	Groups	N	Mean	SD	Test statistic (*t*)	Significance (*p*)
552 Hz	Non-musicians	40	4.30	5.00	0.32	0.746
	Musicians	38	3.72	5.91		
781 Hz	Non-musicians	40	9.85	8.19	0.76	0.450
	Musicians	38	7.72	8.98		
1104 Hz	Non-musicians	40	17.85	6.90	0.96	0.340
	Musicians	38	15.00	10.98		
1563 Hz	Non-musicians	40	25.25	6.53	-0.08	0.932
	Musicians	38	25.44	7.46		
2207 Hz	Non-musicians	40	25.70	4.39	-0.36	0.721
	Musicians	38	26.28	5.49		
3125 Hz	Non-musicians	40	26.80	5.00	-1.16	0.250
	Musicians	38	28.78	5.42		
4419 Hz	Non-musicians	40	25.90	5.47	-1.49	0.144
	Musicians	38	28.67	5.93		
6250 Hz	Non-musicians	40	26.00	9.87	0.16	0.867
	Musicians	38	25.50	8.19		

Supplementary Material S3

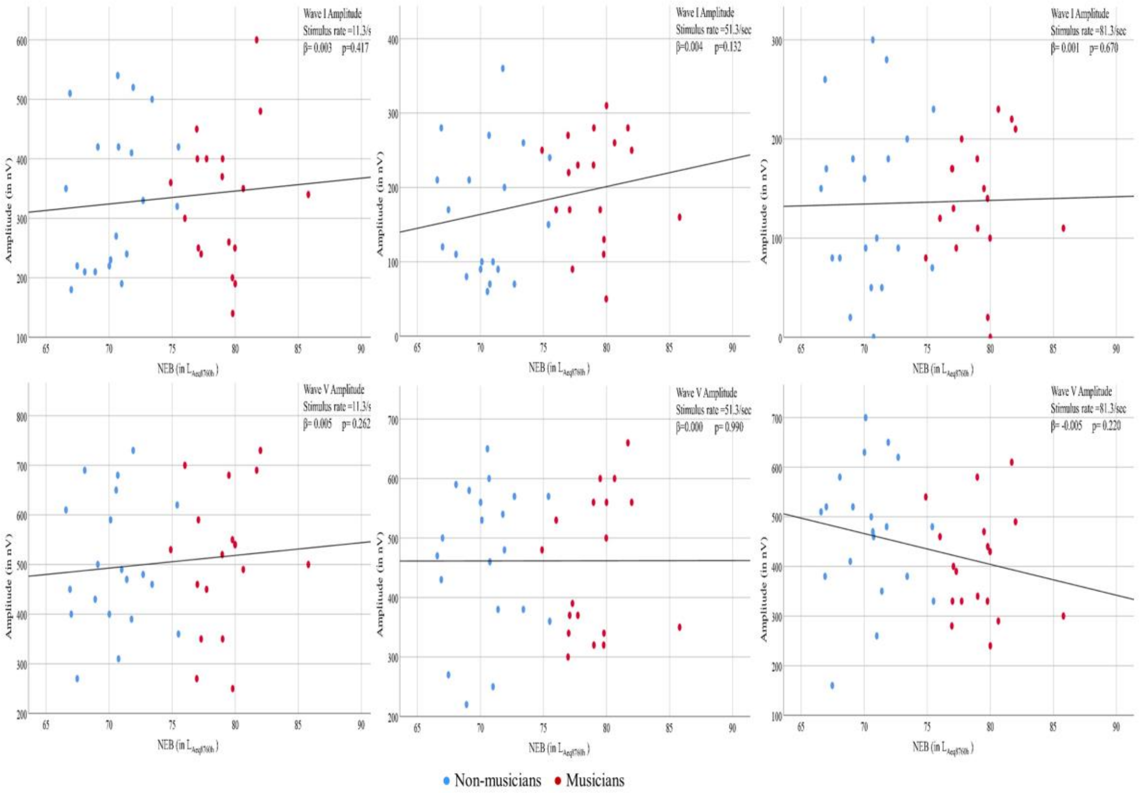

Figure S1. Scatter plots for NEB and ABR wave I amplitude (top row) and NEB and ABR wave V amplitude (bottom row). Linear regression lines were inserted to show the predictive relationship. The stimulus repetition rate is denoted in each panel (11.3, 51.3, and 81.3/sec). Regression results are shown in each panel.

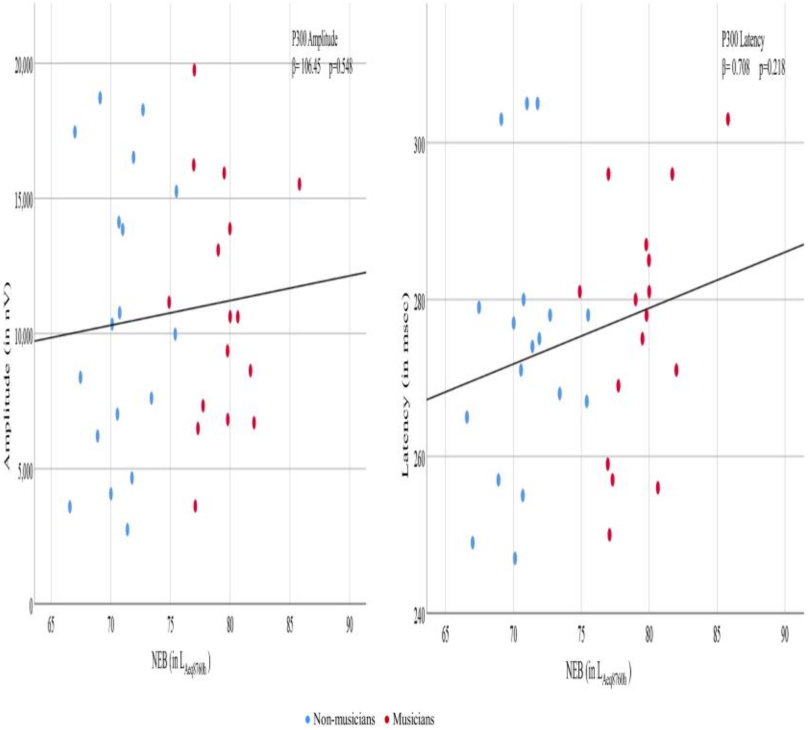

Figure S2. Scatter plots for NEB and P300 amplitude (left panel) and NEB and P300 latency (right panel). Linear regression lines were inserted to show the predictive relationship

Supplementary Material S4

Summary of the mixed effect linear regression analysis : main effects for non-musician/musician groups, gender, groups-gender interaction, groups-SNRs interaction, and gender- SNR interaction on CNC test.

Effect	Num DF	Den DF	F Value	Sig.
Groups	1	34	8.630	**0.006**
Gender	1	34	1.623	0.211
SNRs	4	140	526.737	0.000
Group*Gender	1	34	0.772	0.386
Gender*SNRs	4	140	0.210	0.932
Groups*SNRs	4	140	0.684	0.604

Summary of the mixed effect linear regression analysis: main effects for non-musician/musician groups, gender, groups-gender interaction, groups-SNRs interaction, and gender- SNR interaction on AzBio test.

Effect	Num DF	Den DF	F Value	Sig.
Group	1	34	0.302	0.586
Gender	1	34	0.082	0.776
SNR	4	140	1002.669	0.000
Group*Gender	1	34	0.310	0.581
Gender*SNR	4	140	0.763	0.551
Group*SNR	4	140	1.268	0.285
